# Single-Pulse Transcranial Magnetic Stimulation-Evoked Potential Amplitudes and Latencies in the Motor and Dorsolateral Prefrontal Cortex among Young, Older Healthy Participants, and Schizophrenia Patients

**DOI:** 10.3390/jpm11010054

**Published:** 2021-01-17

**Authors:** Yoshihiro Noda, Mera S. Barr, Reza Zomorrodi, Robin F. H. Cash, Pantelis Lioumis, Robert Chen, Zafiris J. Daskalakis, Daniel M. Blumberger

**Affiliations:** 1 Department of Neuropsychiatry, Graduate School of Medicine, Keio University School of Medicine, Tokyo 160-8582, Japan; 2 Department of Psychiatry, University of Toronto, Toronto, ON M5T 1R8, Canada; mera.barr@gmail.com (M.S.B.); Daniel.Blumberger@camh.ca (D.M.B.); 3 Temerty Centre for Therapeutic Brain Intervention, Centre for Addiction and Mental Health, Toronto, ON M6J 1H4, Canada; Reza.Zomorrodi@camh.ca; 4 Monash Alfred Psychiatry Research Centre, Monash University Central Clinical School and the Alfred, Melbourne 3004, Australia; robin.cash2@gmail.com; 5 Department of Neuroscience and Biomedical Engineering, Aalto University School of Science, FI-00076 Espoo, Finland; plioumis@gmail.com; 6 Division of Neurology, Department of Medicine, University of Toronto, Division of Brain, Imaging and Behaviour—Systems Neuroscience, Krembil Research Institute, University Health Network, Toronto, ON M5T 2S8, Canada; robert.chen@uhn.ca; 7 Department of Psychiatry, UC San Diego Health, San Diego, CA 92093, USA; zdaskalakis@health.ucsd.edu; 8 Campbell Family Mental Health Research Institute, Centre for Addiction and Mental Health, Toronto, ON M5T 1R8, Canada

**Keywords:** TMS-EEG, TMS-evoked potentials, dorsolateral prefrontal cortex, motor cortex, schizophrenia

## Abstract

Background: The combination of transcranial magnetic stimulation (TMS) with electroencephalography (EEG) allows for non-invasive investigation of cortical response and connectivity in human cortex. This study aimed to examine the amplitudes and latencies of each TMS-evoked potential (TEP) component induced by single-pulse TMS (spTMS) to the left motor (M1) and dorsolateral prefrontal cortex (DLPFC) among healthy young participants (YNG), older participants (OLD), and patients with schizophrenia (SCZ). Methods: We compared the spatiotemporal characteristics of TEPs induced by spTMS among the groups. Results: Compared to YNG, M1-spTMS induced lower amplitudes of N45 and P180 in OLD and a lower amplitude of P180 in SCZ, whereas the DLPFC-spTMS induced a lower N45 in OLD. Further, OLD demonstrated latency delays in P60 after M1-spTMS and in N45-P60 over the right central region after left DLPFC-spTMS, whereas SCZ demonstrated latency delays in N45-P60 over the midline and right central regions after DLPFC-spTMS. Conclusions: These findings suggest that inhibitory and excitatory mechanisms mediating TEPs may be altered in OLD and SCZ. The amplitude and latency changes of TEPs with spTMS may reflect underlying neurophysiological changes in OLD and SCZ, respectively. The spTMS administered to M1 and the DLPFC can probe cortical functions by examining TEPs. Thus, TMS-EEG can be used to study changes in cortical connectivity and signal propagation from healthy to pathological brains.

## Highlights

- The single-pulse transcranial magnetic stimulation (TMS) (spTMS) allows probing and visualization of the spatiotemporal characteristics of each young (YNG), older (OLD), and schizophrenia (SCZ) group.
- Different TMS-evoked potential (TEP) characteristics induced by spTMS were observed in the OLD and SCZ compared with YNG.
- Specifically, changes in P30 and N45 deflections of TEPs by spTMS were important in classifying these groups across the motor cortex (M1) and dorsolateral prefrontal cortex (DLPFC).

## 1. Introduction

Electroencephalography (EEG) studies probed by transcranial magnetic stimulation (TMS) have become feasible since Ilmoniemi et al. initially developed the technology of the TMS-compatible EEG system [1]. One advantage of the TMS-EEG paradigm is that it allows for the investigation and mapping of cortical regions without the need to administer perception stimuli (e.g., visual, auditory, sensory stimuli) or specific cognitive tasks as in event-related potential studies. The TMS-EEG technique has been applied in several research labs worldwide mainly for healthy participants [2,3,4,5,6,7,8], and previous studies using TMS-EEG have demonstrated the typical pattern of TMS-evoked potentials (TEPs) and their topographical distribution [9,10,11,12]. Specifically, single-pulse TMS (spTMS) to the primary motor cortex (M1) as well as the dorsolateral prefrontal cortex (DLPFC) induces a well-characterized series of TEP components of P30, N45, P60, N100, and P180 [9,10,13], that are highly reproducible [10] and the amplitudes of these TEP components vary depending on the intensity of TMS [14,15]. Individual TEP components are thought to be mediated by distinct inhibitory and excitatory processes. Moreover, it has been reported that amplitudes of TEPs elicited by spTMS vary depending on the stimulated area [16], notable being reduced at the DLPFC compared to M1-spTMS [17,18].

A previous spTMS-EEG study investigated the topographical distribution pattern and amplitude power of TEPs after left superior frontal cortex stimulation between healthy young (YNG) and older (OLD) participants compared to patients with Alzheimer’s disease [19]. The study demonstrated no significant difference in the topographical distribution of TEPs or amplitude power of early TEP components (N45 to N100) between healthy YNG and OLD participants. However, significantly smaller distribution area and power of the early TEPs were observed among patients with Alzheimer’s disease compared to healthy young participants. The other spTMS-EEG studies examined spectrum power and time-frequency analyses of TEPs after premotor cortex stimulation between patients with schizophrenia (SCZ) and healthy participants, which demonstrated a significantly smaller early TEP power [20] and reduced amplitude of TMS-induced gamma oscillations and its effective connectivity [21] in patients with SCZ compared to the healthy participants.

In OLD adults as well as in patients with SCZ, it is known that atrophy of the cerebral cortex is associated with aging [22] or disease progression [23]. Further, age and neuropathological changes may contribute to decreased nerve conduction velocity [24,25,26]. Therefore, it is speculated that TEP responses by spTMS, especially in latency, may be reduced in these populations compared to younger healthy adults. Thus, it may be possible to probe these physiological and pathological alterations using spTMS-EEG [27].

To our knowledge, there are a limited number of studies [19,20,21,28] examining the spatiotemporal patterns of TEP components by spTMS in OLD adults and patients with SCZ compared to YNG adults, specifically investigating the TEPs with stimulation of the DLPFC, which is an area more directly associated with the changes that occur during healthy aging as well as the pathophysiology of SCZ [29,30]. It is therefore important to identify the neurophysiological profile of TEPs by spTMS among healthy and pathological brains to better understand the pathophysiology of neuropsychiatric disorders [31,32]. Based on the previous findings that showed reduced TEP amplitude with age [19,20,21,28], we hypothesized that the OLD group and SCZ group would have significantly lower amplitudes of TEPs and more delayed latencies of TEPs compared to those of the YNG group. Thus, we aimed to investigate the amplitudes and latencies of TEPs by spTMS to M1 as well as the DLPFC in an exploratory fashion from both the stimulated region (i.e., M1 and DLPFC) and other connected regions of interest (ROI: contralateral M1, DLPFC, and midline central area) among the groups (YNG, OLD, and SCZ).

## 2. Materials and Methods

### 2.1. Participants

Twelve right-handed YNG (6 female, mean age: 39 ± 12 yrs), 12 right-handed OLD (6 female, mean age: 72 ± 9 yrs), and 12 right-handed patients with SCZ (4 female, mean age: 41 ± 10 yrs) participated in this study. Participants of all groups were eligible to participate in this study if they met the following criteria: (i) between ages 18 and 59 for the YNG and SCZ, or ages above 60 for the OLD; (ii) no history of neurological disorders including seizure or stroke; (iii) no history of alcohol or other drug abuse/dependence; and (iv) not being a smoker.

In addition, the YNG and OLD also satisfied with the following criteria: (v) no history of neuropsychiatric disorders; (vi) normal cognitive function assessed by neurocognitive battery; (vii) no prescription medications; while patients with SCZ met the following criteria; (viii) not taking benzodiazepines and anticholinergics. All participants were screened with the Structured Clinical Interview for DSM–IV Axis I Disorders or the Mini-International Neuropsychiatric Interview prior to study participation. The present study was performed according to the Declaration of Helsinki and was reviewed and approved by the Research Ethics Board of the Centre for Addiction and Mental Health.

### 2.2. TMS Procedure

Monophasic TMS pulses were administered to the left M1 using a 70 mm figure-of-eight coil, and a Magstim 200 stimulator (Magstim Company Ltd., Whitland, UK). During the TMS testing, the participants sat in a chair with their eyes open, and their bodies relaxed throughout the study. First, the M1 hotspot site for the right first dorsal interosseous muscle to evoke the largest motor evoked potential (MEP) with the lowest intensity was determined. Second, the individual intensity to induce 1 mV peak-to-peak MEP amplitude of the same muscle was determined. The intensity to induce 1 mV peak-to-peak MEP amplitude was used for the spTMS in this study. At each stimulation site of M1 and DLPFC, 100 TMS pulses were applied per session, respectively. In addition, the stimulation site of the left DLPFC was identified using the EEG cap method. Specifically, the F5 electrode site was used as the target site of the left DLPFC according to our other studies (Noda et al., 2017a, Noda et al., 2017b).

### 2.3. EEG Recording and Pre-Processing

EEG was recorded through a 64-channel Neuroscan Synamps 2 with a TMS-compatible EEG cap (Compumedics Neuroscan, Victoria, Australia). Recording electrodes impedance was kept below 5 kΩ during the experiment. All electrodes were referenced to an electrode placed on the vertex. EEG signals were recorded at DC with a sampling rate of 20 kHz and then an online lowpass filter of 200 Hz was applied. EEG data were processed offline using the MATLAB software (R2014a, The MathWorks, Natick, MA, USA).

### 2.4. EEG Signal Processing

EEG signal processing was performed in accordance to already published methodology (Noda et al., 2016). All EEG data were epoched from −1000 ms to 2000 ms relative to the TMS pulse. Baseline correction was performed with respect to the pre-stimulus interval −500 ms to −110 ms. The epoched EEG data was re-segmented from 10 ms to 2000 ms post-TMS to limit the TMS-induced artifact. Then, EEG data were visually inspected to exclude trials and channels that were highly contaminated with noise. Subsequently, independent component analysis (ICA) was applied to minimize and remove the typical TMS-related decay artifacts as well as eye-related and muscle activity related components. Next, the Butterworth, zero-phase shift 1–55 Hz bandpass filter (24 dB/Oct) and notch filter were applied. Then, the processed data were downsampled to 1000 Hz. In each subject, the number of ICA components that were removed from the original 62 ICA components was no greater than 20%. Finally, data were re-referenced to the average reference for further analyses.

### 2.5. Single-Pulses TMS-Evoked Potential (TEP) Analyses

**Figure 1:**
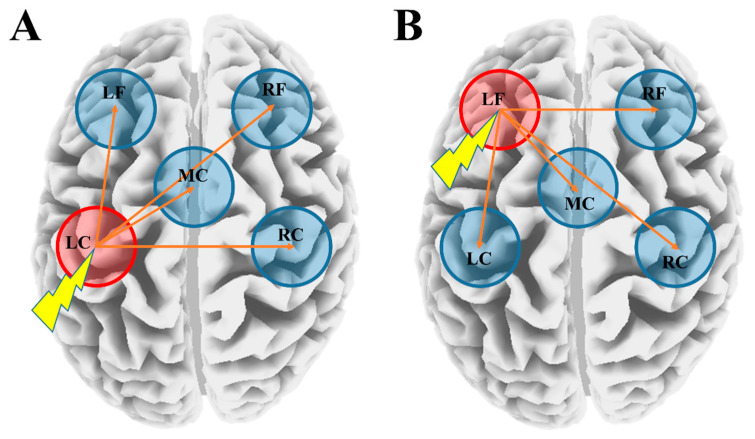
Schematic diagram of the transcranial magnetic stimulation (TMS) stimulation site and analysis site. (**A**) depicts single-pulse TMS (spTMS) administered to the left motor cortex (M1), while (**B**) shows spTMS applied to the left dorsolateral prefrontal cortex (DLPFC).

The TEP was analyzed individually focusing on the amplitude and latency of each TEP component (P30, N45, P60, N100, and P180) at the 5 ROIs (left frontal: AF3, F3, F5; right frontal: AF4, F4, F6; left central: C3, C5, CP5; right central: C4, C6, CP6; midline central: Fz, FCz, Cz; see Figure 1), which were obtained from M1-spTMS and DLPFC-spTMS experiments.

### 2.6. Statistical Analyses

The SPSS Statistics 19 (IBM, Armonk, New York, USA) was used for statistical analysis. In this study, normal distributions of the TEP data were confirmed with the Shapiro-Wilk test before performing the parametric statistical testing. For the analyses of TEP amplitudes, the analysis of variance (ANOVA) with TEP components (i.e., P30, N45, P60, N100, and P180) as a within-subject factor and groups (i.e., YNG, OLD, or SCZ) as a between-subject factor was applied for each M1-spTMS and the DLPFC-spTMS paradigm, separately. Here, we analyzed TEP data of M1-spTMS focusing on the left central (LC) ROI (i.e., left M1) while we analyzed TEP data of the DLPFC-spTMS focusing on the left frontal (LF) ROI (i.e., left DLPFC). When there was a significant interaction between the TEP component and group, post-hoc independent t-tests were applied to examine group differences of TEP amplitudes. Next, we conducted the multiple regression analysis with the group (i.e., YNG, OLD, or SCZ) as a dependent variable and the site of stimulation (i.e., M1 or DLPFC) and TEP component (i.e., P30, N45, P60, N100, and P180) as independent variables to explore the predictive factors to identify the groups using an enter method. In this regression analysis, we set a significant level of α = 0.05/10 (site of stimulations*TEP components) with Bonferroni correction.

For the analyses of TEP latencies, one-way ANOVAs and post-hoc paired t-tests were applied to explore the group difference of each TEP component for the same ROIs. Further, within-group level, the differences of TEP latencies between the LC ROI (i.e., the stimulated region of M1-spTMS) and other ROIs for M1-spTMS as well as between the LF ROI (i.e., the stimulated region of the DLPFC-spTMS) and other ROIs for the DLPFC-spTMS. A significant level of α = 0.05/25 (site of ROIs*TEP components) with Bonferroni correction was applied here.

## 3. Results

### 3.1. TEP Amplitude Differences between the YNG, OLD, and SCZ Groups

**Figure 2:**
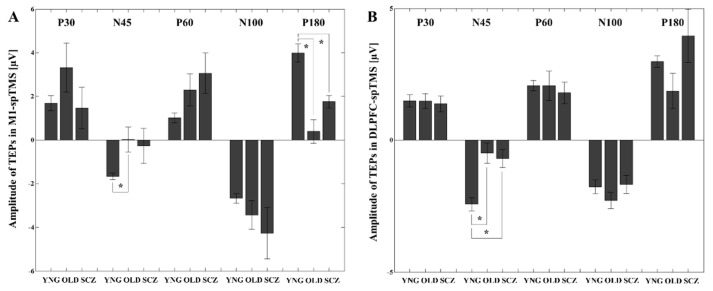
Amplitude differences for each TMS-evoked potential (TEP) component among healthy young (YNG), older (OLD), and patients with schizophrenia (SCZ) at the left central (LC) ROI (left M1). TEP amplitude differences among the groups at the left M1 induced by M1-spTMS were demonstrated in (**A**), while TEP amplitude differences among the groups over the left DLPFC induced by the DLPFC-spTMS are shown in (**B**). Compared to the YNG group, the OLD group had a significantly lower amplitude of the N45 TEP, while the OLD group and SCZ group had significantly lower amplitudes on the P180 TEP over the LC ROI (left M1) in M1-spTMS paradigm. In the DLPFC-spTMS, compared to the YNG group, the OLD group and SCZ group had significantly lower amplitudes on N45 TEP over the left frontal ROI (left DLPFC). * Significant differences.

With M1-spTMS, at the LC ROI (i.e., left M1), the ANOVA indicated a significant TEP-by-group interaction (F_8,132_ = 4.504, *p* < 0.0001). Subsequently, compared to the YNG group, post-hoc independent t-tests revealed that the OLD group had significantly lower amplitudes of N45 (t_22_ = −2.872, *p* = 0.009; amplitude N45 at the LC in YNG > amplitude N45 at the LC in OLD) and P180 (t_22_ = 5.283, *p* < 0.0001; amplitude P180 at the LC in YNG > amplitude P180 at the LC in OLD) TEPs, whereas the SCZ group had a significantly lower amplitude of P180 (t_22_ = 4.394, *p* = 0.0002; amplitude P180 at the LC in HC > amplitude P180 at the LC in SCZ). The results of TEPs after M1-spTMS are depicted in Figure 2A.

On the other hand, at the LF ROI (i.e., left DLPFC) by the DLPFC-spTMS, the ANOVA showed a significant TEP-by-group interaction (F_8,132_ = 2.599, *p* = 0.011). Post-hoc independent t-tests indicated that the OLD group had a significantly lower amplitude of N45 (t_22_ = −4.200, *p* = 0.0004; amplitude N45 at the LF in YNG > amplitude N45 at the LF in OLD), while the SCZ group also had a significantly lower amplitude of N45 (t_22_ = −4.047, *p* = 0.001; amplitude N45 at the LF in HC > amplitude N45 at the LF in SCZ), compared to the YNG group. The results of TEPs by the DLPFC-spTMS are described in Figure 2B.

Regarding P30 and N100 TEP amplitudes, no significant differences were observed among the three groups both in M1-spTMS or DLPFC-spTMS paradigm

Representative TEP waveforms from the left DLPFC obtained by spTMS for the YNG, OLD, and SCZ groups in this study are shown in the Supplementary Figure S1.

### 3.2. Predictive Factors to Classify the Groups Based on the Multiple Regression Analysis 

The multiple regression analysis revealed that P30 and N45 components were significant factors (P30: β = −0.410, *p* = 0.004; N45: β = 6.22, *p* < 0.001; F6, 65 = 4.825, adjusted R^2^ = 0.244) to identify the difference between these groups. 

### 3.3. Latency Differences for Each TEP between the YNG, OLD, and SCZ Groups

**Figure 3:**
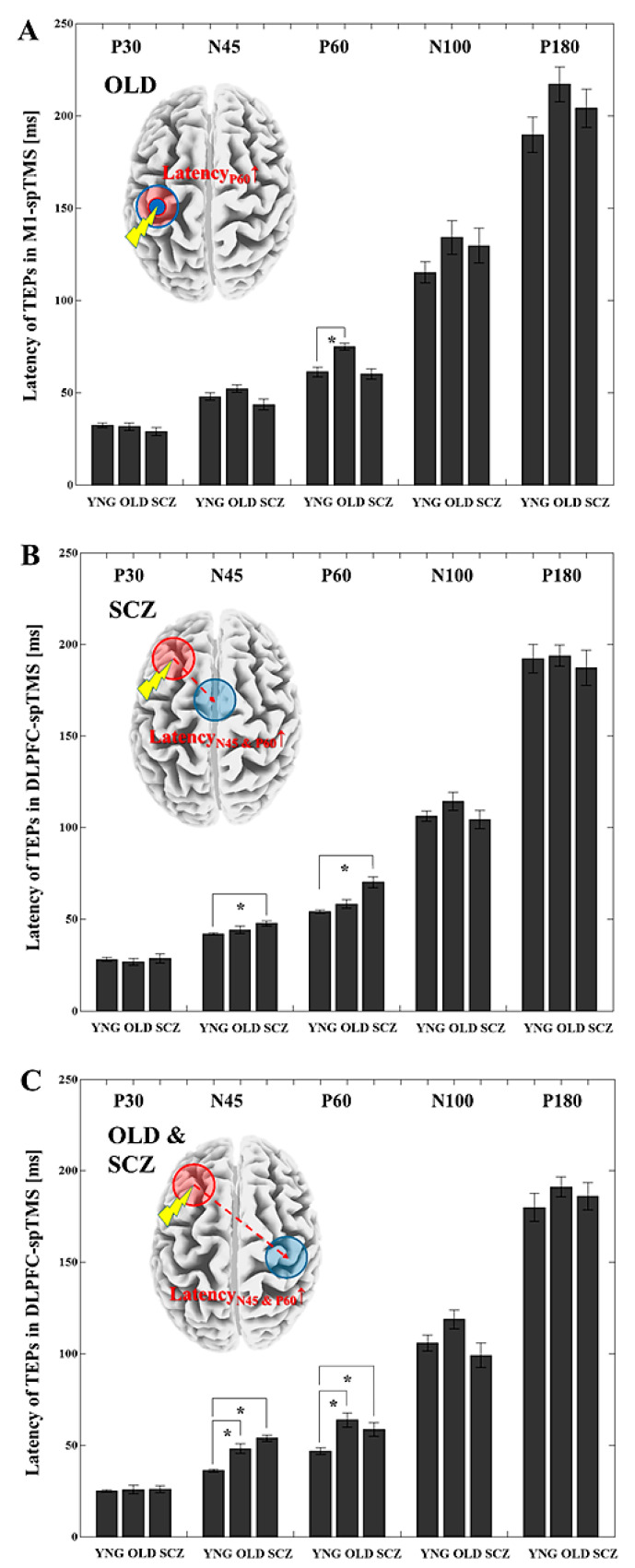
Latency differences for each TEP component among healthy young (YNG) participants, older (OLD) participants, and patients with schizophrenia (SCZ). TEP latency differences among groups at the LC ROI (left M1) induced by M1-spTMS were demonstrated in (**A**), while TEP latency differences among the groups induced by the DLPFC-spTMS are shown in (**B**) (over the midline central ROI) and (**C**) (over the right central ROI). In M1-spTMS, compared to the YNG group, the OLD group showed a significantly longer latency of the N45 TEP over the left M1 in M1-spTMS paradigm, whereas in the DLPFC-spTMS, the SCZ group showed significantly longer latencies of the N45 and P60 TEPs over the midline central ROI. Furthermore, after DLPFC-spTMS, the OLD group and SCZ group demonstrated significantly longer latencies of N45 and P60 TEPs over the right M1. In (**A**–**C**), a schematic diagram of a group showing significant findings is shown in the box. * Significant differences.

With M1-spTMS, when we compared each TEP latency over all 5 ROIs between the YNG and OLD groups, the OLD group had a significantly longer P60 latency (t_22_ = −4.275, *p* = 0.0003; latency P60 at the LC in YNG < latency P60 at the LC in OLD) over the LC ROI compared to the YNG group (see Figure 3A).

Likewise, when we looked at the DLPFC-spTMS paradigm, the OLD group showed significantly longer N45 (t_22_ = −4.255, *p* = 0.0003) and P60 (t_22_ = −4.033, *p* = 0.0006) TEP latencies (latencies N45-P60 at the RC in YNG < latencies N45-P60 at the RC in OLD) from the right central ROI compared to the YNG group. The SCZ group indicated significantly longer N45 (t_22_ = −3.622, *p* = 0.0015) and P60 (t_22_ = −5.095, *p* < 0.0001) TEP latencies (latencies N45-P60 at the MC in HC < latencies N45-P60 at the MC in SCZ) at the midline central ROI (Figure 3B) as well as longer N45 (t22 = −9.207, *p* < 0.0001) and P60 (P60: t_22_ = −2.945, *p* = 0.007) TEP latencies (latencies N45-P60 at the RC in HC < latency N45-P60 at the RC in SCZ) at the right central ROI (Figure 3C). For P30 and N100 TEP latencies, there were no significant differences among groups both in M1-spTMS or DLPFC-spTMS paradigm.

### 3.4. Latency Differences between the Stimulated ROI and Other ROIs within the YNG, OLD, and SCZ Groups

**Figure 4:**
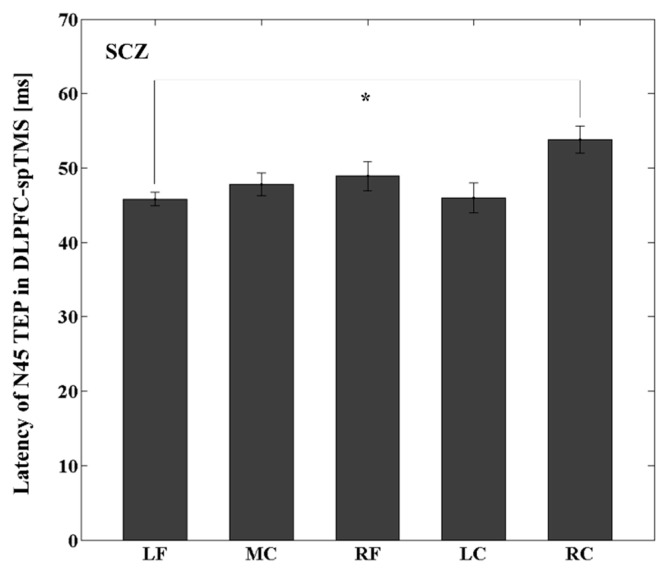
Latency differences between the left DLPFC and other ROIs within a schizophrenia (SCZ) group in the DLPFC-spTMS paradigm. There were no significant TEP latency differences between the left DLPFC and other ROIs within the young (YNG) and older (OLD) groups. However, within the SCZ group, the latency of N45 TEP was significantly longer over the right M1 compared to the left DLPFC. * Significant differences.

With M1-spTMS, there was no significant latency difference between the LC ROI (i.e., left M1) and other ROIs within groups. After DLPFC-spTMS, there was no significant difference in latencies between the LF ROI (i.e., left DLPFC) and other ROIs within the YNG or OLD group. However, within the SCZ group, there was a significant latency difference on N45 (t_11_ = −4.326, *p* = 0.0012; latency N45 at the LF in SCZ < latency N45 at the RC in SCZ) between the LF (i.e., left DLPFC) and right central ROIs (i.e., right M1), showing that the latency of N45 was significantly longer from the right central ROI compared to that of the left DLPFC (see Figure 4).

## 4. Discussion

The present study demonstrated unique spatiotemporal profiles of TEPs induced by spTMS of M1 and DLPFC. First, the OLD group demonstrated an attenuated response in the amplitude of N45 after M1-spTMS, which is in line with previous studies showing that there was a TEP amplitude reduction with age in healthy subjects [21,28], compared to the YNG group. Further, both the OLD and SCZ groups had attenuated responses in the amplitude of P180 after M1-spTMS, which is, at least in part, consistent with a previous study showing that SCZ had reduced spTMS-evoked gamma oscillations [21], as well as the amplitude of N45 by the DLPFC-spTMS, compared to those of the YNG group. Second, the OLD group showed a delayed response in the latency of P60 at the stimulated ROI after M1-spTMS compared to the YNG group. The SCZ group demonstrated a delayed response in the latency of N45 over the right central ROI within-subjects level as well as delayed responses in the latency of N45-P60 at the midline central ROI between-subjects level by the DLPFC-spTMS. In addition, the OLD and SCZ groups demonstrated a delayed response in the latency of N45-P60 over the right central ROI after DLPFC-spTMS compared to the YNG group.

To date, the origin and mechanisms of N45 and P180 have not been fully elucidated. However, previous studies demonstrated that N45 may be associated with gamma-aminobutyric acid receptor A (GABA_A_) mediated inhibition [7,33,34,35]. In the healthy YNG group, the amplitude of N45 component was relatively increased consistently across M1 and DLPFC by spTMS in our study (Figure 2). In contrast, P180 has been proposed to relate either to a late excitatory potential or cortical disinhibition. The latter interpretation may be more consistent with the GABAergic deficits shown in both aging and SCZ [29,30]. Thus, it seems that N45 may represent GABA_A_ergic inhibitory function and P180 may reflect more GABA_A_ergic disinhibition rather than just an excitatory potential itself. 

In addition, several lines of evidence have shown that GABA_A_ receptor-mediated inhibition in M1 as indexed by motor-evoked potentials decreases with age [36,37,38]. Further, magnetic resonance spectroscopic (MRS) studies have demonstrated that OLD participants have a significantly lower level of GABA compared to the young participants [39,40]. Therefore, these MRS findings appear at least partially consistent with TMS neurophysiological studies in OLD participants [29].

On the other hand, in patients with SCZ, GABAergic deficit is supported by neuropathological findings that demonstrate substantial reductions in GABAergic interneurons, GABA-synthesizing enzyme glutamic acid decarboxylase, and GABA-related gene expression in the cortex [41,42,43,44,45,46]. Further, there are several lines of evidence that patients with SCZ had reduced GABA_A_ receptor-mediated inhibition [30,47,48] compared to healthy YNG group. 

Again, as shown in Figure 2A, the amplitude of N45 component of spTMS was smaller in the OLD group and SCZ group (especially significantly lower in the OLD group) than in the YNG group, suggesting a relative decrease in GABA_A_ receptor-mediated neurophysiological function. Thus, the decline in inhibitory neurophysiological function due to age-related changes as well as the pathophysiology of schizophrenia may affect the impairment of coordinated motor skills among motor control [49].

In addition, the multiple regression analysis demonstrated that P30 and N45 components are the significant factors to classify the groups. Interestingly, since P30 and N45 TEP components are supposed to be generated by GABA_A_ receptor-mediated function [7,11,35,50], it is likely that GABA_A_ergic inhibition in the cortex is essential to maintain normal brain function.

Moreover, in terms of the response of TEP latencies after spTMS, since N45 and P60 are thought to be linked to GABA_A_ and GABA_B_ receptor-mediated functions as well as its excitatory function, respectively [13,35], our finding of delayed response of P60 latency at the LC ROI by M1-spTMS in the OLD group may be related to the GABA_B_ergic inhibitory and/or its excitatory dysfunctions due to aging [39,40], whereas the findings of delayed responses of N45 and P60 latencies at the midline central and right central ROIs after DLPFC-spTMS in the SCZ group may be associated with GABA_A_ (i.e., N45) and GABA_B_ (i.e., P60) receptor-mediated inhibitory and/or its excitatory (i.e., P60) dysfunctions [13,35]. Thus, these results might reflect conduction delays in both inhibitory and excitatory circuits, and that together these delayed responses of N45 and P60 might contribute to disrupted network connectivity in SCZ. Specifically, recent studies also suggest that a latency of P60 may be a correlate of neuronal excitability in M1 [13] and the DLPFC [51,52]. This reflects a larger distribution of cortical areas possibly due to more widespread neuropathological involvement related to the illness [43,44,45,46]. Indeed, we observed significantly delayed responses of N45-P60 latencies over the right central ROI even in the OLD group. However, the SCZ group demonstrated abnormal TEP latencies across short cortical distances compared to the OLD group (Figure 3), which may represent greater GABAergic inhibition deficits in the SCZ group.

Further, only the SCZ group showed a significantly delayed response of N45 latency over the right central ROI after DLPFC-spTMS. This finding may indicate more widespread GABA_A_ergic inhibitory dysfunction possibly due to the functional disconnection between the left DLPFC and right central ROI in the SCZ [53]. Consistent with this contention, a previous functional magnetic resonance imaging study in SCZ demonstrated the functional hypoconnectivity in the somatomotor network [53], which may support our above finding of the delayed response of N45 in the SCZ group.

There are some limitations in the present study. First, the number of participants for each group was relatively small, although comparable to that of many previous TMS studies [7,35]. Thus, the findings warrant further research with larger sample sizes. Second, since we did not apply the auditory masking system during the experiments, the effect of auditory evoked potentials due to the conduction of the TMS click sounds on TEPs cannot be completely excluded [54]. Therefore, for future TMS-EEG studies, auditory white noise should be used during TMS stimulation as masking of the TMS click sound. Third, since patients with schizophrenia took medications when they participated in this study, there may be some medication effects on TEPs. Therefore, it would be necessary to more strictly control the influence of medications in the future study.

## 5. Conclusions

The present study demonstrated that TEP responses on amplitude and latency induced by spTMS have potential clinical application as a feasible neurophysiological probe to examine cortical functions in healthy people and patients with neuropsychiatric disorders such as SCZ and to assess the therapeutic effects of various interventions on TEP responses. Further, between the OLD and the SCZ groups, there were similarities in TEP responses in amplitude while there was a difference in TEP latency when looking at the propagation after the DLPFC-spTMS. To this end, our findings warrant further research in a larger sample size as well as in other neuropsychiatric disorders.

## Data Availability

Data sharing not applicable. No new data were created or analyzed in this study. Data sharing is not applicable to this article.

## Supplementary Materials

The following are available online at https://www.mdpi.com/2075-4426/11/1/54/s1, Figure S1: Representative TEP waveforms at the left DLPFC obtained by spTMS for the YNG, OLD, and SCZ groups.

## Abbreviations

SpTMS	single-pulse transcranial magnetic stimulation
M1	primary motor cortex
DLPFC	dorsolateral prefrontal cortex
TEP	TMS-evoked potential
