# Supplementary figures and images for: Single-Pulse Transcranial Magnetic Stimulation-Evoked Potential Amplitudes and Latencies in the Motor and Dorsolateral Prefrontal Cortex among Young, Older Healthy Participants, and Schizophrenia Patients
Source: J Pers Med. 2021 Jan 17;11(1):54. doi: 10.3390/jpm11010054 (PMC7830964; doi:10.3390/jpm11010054)

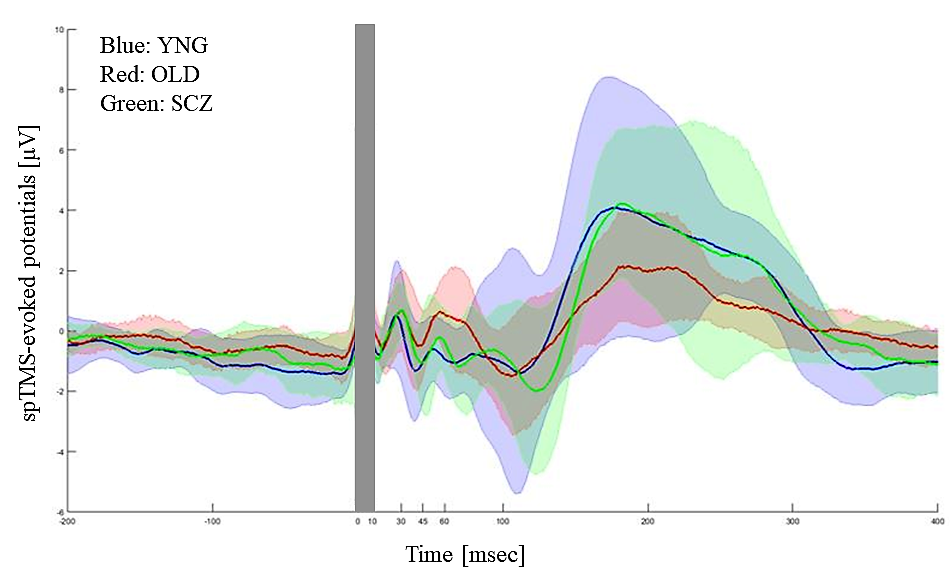

Supplement: Supplementary file 1 [file jpm-11-00054-s001.zip › jpm-1063009-supplementary.tif]
